# Supplementary figures and images for: High quality transcriptome profiling confirms the transcriptional landscape of Treponema pallidum subsp. pallidum
Source: Sci Rep. 2025 Jul 2;15:23272. doi: 10.1038/s41598-025-06583-9 (PMC12223175; doi:10.1038/s41598-025-06583-9)

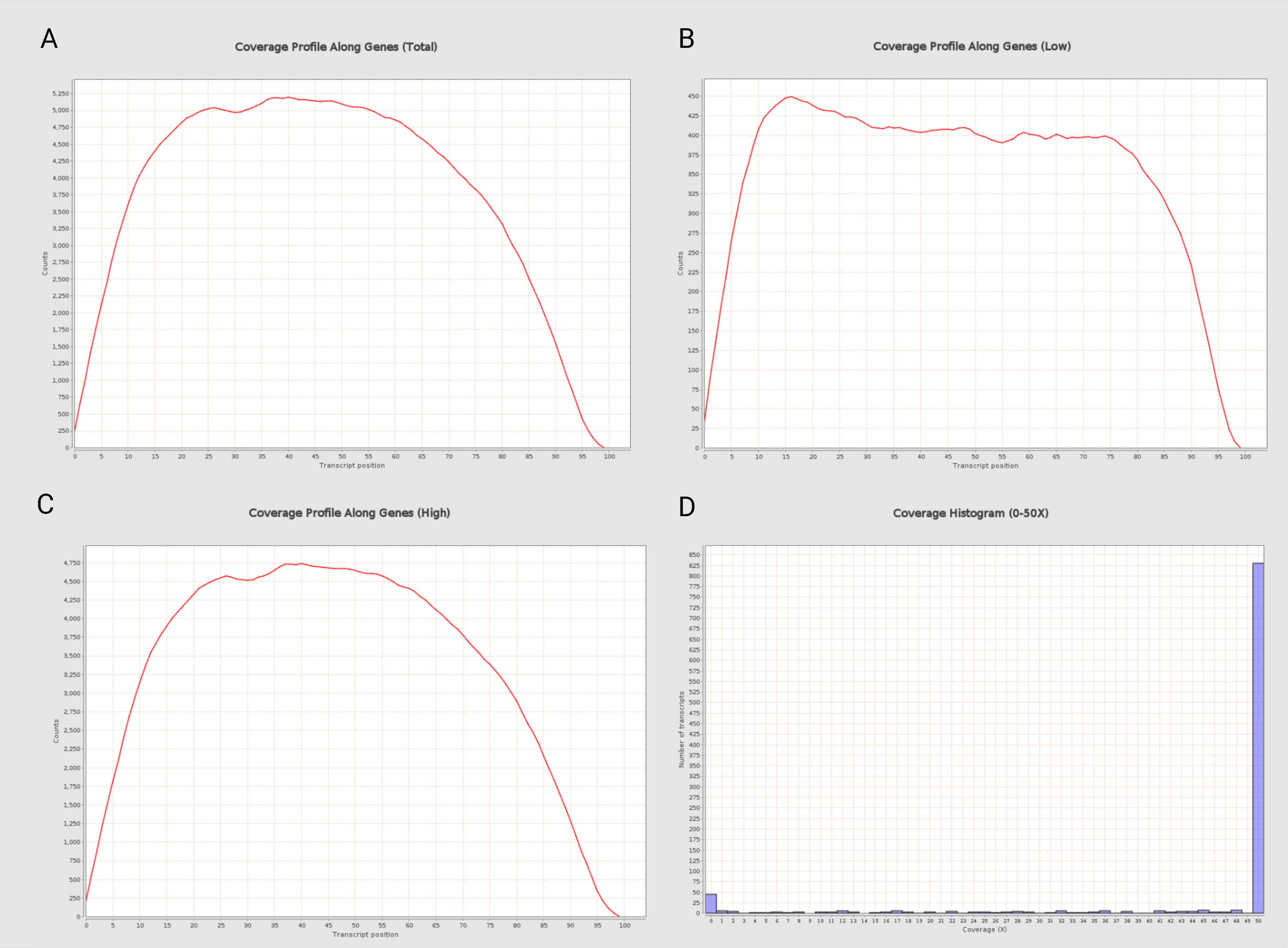

Supplement: Supplementary file 2 — Supplementary Material 2 [file 41598_2025_6583_MOESM2_ESM.jpeg]
