# Supplementary material for: High quality transcriptome profiling confirms the transcriptional landscape of Treponema pallidum subsp. pallidum
Source: Sci Rep. 2025 Jul 2;15:23272. doi: 10.1038/s41598-025-06583-9 (PMC12223175; doi:10.1038/s41598-025-06583-9)
Supplement: Supplementary file 4 — Supplementary Material 4 [file 41598_2025_6583_MOESM4_ESM.docx]

**Supporting information captions**

**S1 Table. SNVs and protein effects found in the genomic sequence of TPA strain Nichols**

**S2 Table. Paralogous regions with ambiguous mapping**

**S3 Table. Annotated region with no evidence of transcription**

**S4 Table. Genes in NC_021490.2 genomic reference coding for hypothetical proteins**

**S5 Table. Potential vaccine candidates**

**S6 Table. Strand-specific transcription tracks details for all annotated regions of the genome and all intergenic regions larger than 10 bp**

**S7 Table. Regions with antisense transcript originating within annotated gene**

**S8 Table. Regions transcribed exclusively in the opposite orientation to the original annotation**

**S9 Table. Regions with transcripts arising from the intergenic region**

**S10 Table. Transcripts with open reading frames corresponding to annotations found in other *Treponema* spp. genomes but absent in NC_021490.2**

**S11 Table. Operons identified in TPA genome**

**S1 Figure. Qualimap Report.A.** Coverage profile along genes (total). **B.** Coverage profile along genes (low).**C.** Coverage Profile along genes (high).**D.** Coverage histogram.

**S1 File. TPA Nichols annotation reference**
